# The Current Landscape of Antibiotic Resistance of *Salmonella* Infantis in Italy: The Expansion of Extended-Spectrum Beta-Lactamase Producers on a Local Scale

**DOI:** 10.3389/fmicb.2022.812481

**Published:** 2022-03-28

**Authors:** Lisa Di Marcantonio, Romina Romantini, Francesca Marotta, Alexandra Chiaverini, Katiuscia Zilli, Anna Abass, Elisabetta Di Giannatale, Giuliano Garofolo, Anna Janowicz

**Affiliations:** ^1^Bacteriology and Diary Production Hygiene Department, Istituto Zooprofilattico Sperimentale dell’Abruzzo e del Molise “G. Caporale,” Teramo, Italy; ^2^Hygiene in Food Technology and Animal Feeds, Istituto Zooprofilattico Sperimentale dell’Abruzzo e del Molise “G. Caporale,” Teramo, Italy

**Keywords:** *Salmonella* Infantis, ESBL–extended-spectrum beta-lactamase, multidrug resistance, genomics, WGS–whole-genome sequencing

## Abstract

*Salmonella enterica* serovar Infantis is one of the five main causes of human salmonellosis in the European Union (EU) and in recent years, has been increasingly reported to carry multiple antimicrobial resistance determinants, including extended-spectrum beta-lactamase (ESBL) genes. In our study, we used WGS-based tools to characterize *S.* Infantis strains circulating in the Abruzzo and Molise regions of Italy between 2017 and 2020 and compared this local dataset to the *S.* Infantis population present in Italy over the last two decades. Phylogenetic analyses demonstrated that the majority of strains isolated from poultry and turkeys from Abruzzo and Molise were closely related and belonged to one of the two main genetic clusters present in Italy, which were grouped predominantly as ESBL-producing strains that harbored pESI-like plasmid. We showed that 60% of the local strains carried multiple antibiotic resistance genes, including ESBL gene *bla*_CTX–M–1_ as well as *aad*A1, *dfr*A1, *dfr*A14, *sul1*, and *tet*(A) genes present on the pESI-like megaplasmid. The analysis of strains from Abruzzo and Molise and the publicly available Italian *S.* Infantis sequences revealed a dramatic increase in the number of identified AMR genes in the strains isolated after 2011. Moreover, the number of strains resistant to five or more antibiotic classes increased from 20–80% in the last decade likely due to the acquisition of the megaplasmid. The persistence of the ESBL-producing and the multidrug-resistant (MDR) clone of *S.* Infantis in poultry populations in Italy and in Europe requires rapid and efficient intervention strategies to prevent further expansion of the clone.

## Introduction

Non-typhoidal *Salmonella* is one of the main causes of acute enteric diseases world-wide and salmonellosis is the second most frequently notified zoonosis in the European Union (EU) (European Food Safety Authority [EFSA] and European Centre for Disease Prevention and Control [ECDC], 2020, 2021). Among all serovars of *Salmonella*, *Salmonella enterica*, serovar Infantis is one of the five main serovars responsible for human infections in the EU and the primary cause of salmonellosis acquired by consumption or handling of contaminated poultry meat (Antunes et al., 2016). The rate of salmonellosis reported in Italy in 2019 was 5.4 cases per 100,000 population and 151 outbreaks have been registered (European Food Safety Authority [EFSA] and European Centre for Disease Prevention and Control [ECDC], 2021). Although *S.* Infantis is frequently isolated from poultry meat, to date, only a few outbreaks caused by this serovar were reported in Italy (Chironna et al., 2014). In the EU between 2018 and 2019 *S.* Infantis was most frequently isolated from broiler chickens, both from animals and the related food sources, followed by turkey and turkey meat, and more than 50% of reported *S.* Infantis from broilers were isolated in Italy (European Food Safety Authority [EFSA] and European Centre for Disease Prevention and Control [ECDC], 2020, 2021). Indeed, of all *Salmonella* serovars the proportion of *S.* Infantis isolated in Italy from poultry sources increased from 2.3% in 2008 to 22.7% in 2018 (IZSVenezie, 2008-2018). Importantly, this increase was also associated with a high proportion of *Salmonella* resistant to multiple antimicrobials, including ciprofloxacin and the 3rd-generation cephalosporins (European Food Safety Authority [EFSA] and European Centre for Disease Prevention and Control [ECDC], 2021). Italy is one of the largest consumers of antimicrobials in the EU (European Food Safety Authority [EFSA] and European Centre for Disease Prevention and Control [ECDC], 2021). Biomass-weighted consumption data reported for 2017 showed that the amount of antimicrobial agents used in food-producing animals in Italy was more than double of the average consumption in EU countries (European Medicines Agency [EMA], 2019; European Food Safety Authority [EFSA] and European Medicines Agency [EMA] and European Centre for Disease Prevention and Control [ECDC], and European Medicines Agency [EMA] et al., 2021). The most commonly purchased antimicrobials for veterinary use were penicillins, tetracyclines, and sulphonamides. Similarly, a high number of antimicrobials were acquired for human use making Italy the 6th largest consumer of antimicrobials per population number. Importantly, the use of 3rd and 4th level cephalosporins in humans was higher than in all EU countries except in Bulgaria (European Food Safety Authority [EFSA] and European Medicines Agency [EMA] and European Centre for Disease Prevention and Control [ECDC], and European Medicines Agency [EMA] et al., 2021).

Global dissemination of MDR strains of *Salmonella* and other Enterobacteriaceae and, in particular, the emergence of extended spectrum beta-lactamase (ESBL)-producing strains, has been increasingly observed in the last two decades. This has prompted the World Health Organization (WHO) to place the ESBL-producing Enterobacteriaceae on the list of “Critical Priority Pathogens” that pose a great risk to public health (World Health Organization [WHO], 2019). MDR strains of *S.* Infantis isolated in broilers have been now recorded in multiple countries, including Hungary (Szmolka et al., 2018), Germany (García-Soto et al., 2020), Italy (Alba et al., 2020; Proietti et al., 2020), the Netherlands (Mughini-Gras et al., 2021), Russia (Bogomazova et al., 2020), and United States (Tate et al., 2017). A recent study showed that global population of *S.* Infantis could be divided into three major lineages (Gymoese et al., 2019). The main lineage, which separated from the other branches approximately 75 years ago, contained the highest number of strains currently circulating worldwide. Several clusters of *S.* Infantis were reported in Europe. Most of these clusters were not geographically restricted and the same clones were isolated in multiple European countries likely due to the shared livestock suppliers (Alba et al., 2020).

An important factor for the increased prevalence of this serovar and the observed MDR has been the acquisition of the pESI-like conjugative megaplasmid, first described in an Israeli strain of *S.* Infantis (Aviv et al., 2014) and later identified worldwide (Franco et al., 2016; Alba et al., 2020; McMillian et al., 2020). This plasmid (∼300 kbp in size) was demonstrated to harbor virulence factor genes such as fimbriae (*ipf* and *fea*), yersiniabactin operon (*ybt*), toxin/antitoxin system, genes conferring resistance to heavy metals (*mer*A), disinfectants (*qac*EΔ), and several antimicrobial resistance genes, including *bla*_CTX–M_ genes responsible for production of ESBL enzymes (Aviv et al., 2014; Alba et al., 2021).

Following the global trend, an increased number of *S.* Infantis, isolated primarily from poultry and turkeys and resistant to several classes of antibiotics, including the 3rd generation cephalosporins, has been observed in the Abruzzo and Molise regions of Italy over the last 10 years. In this work, we characterized the *S.* Infantis population found in these regions and placed the local dataset in a broader phylogeny generated for the entirety of Italy. We analyzed *S.* Infantis isolated between 2017 and 2020 during passive surveillance activities of the Regional Reference Laboratory which collects and characterizes pathogenic Enterobacteria data from the Abruzzo and Molise regions as part of a national network of surveillance. We used whole-genome sequencing (WGS)-based tools to identify genomic traits leading to multidrug resistance of the locally isolated strains of *S.* Infantis and compared them with the Italian dataset. In particular, we aimed to describe the ESBL-producing population and identify the genetic features responsible for observed increase in resistance to the 3rd generation cephalosporins in the strains circulating in the studied regions. The results presented in our study will provide additional data that will aid the Italian surveillance system in making informed decisions and form an important part in the antimicrobial resistance monitoring activities of the network. In particular, the study will provide the most recent genomic data regarding the circulating *S.* Infantis strains and the genetic determinants of the antimicrobial resistance currently found in the studied *S.* Infantis population.

## Materials and Methods

### Isolation and Phenotypic Characterization of Bacterial Strains

Strains were collected during routine activities of the Regional Reference Laboratory for Pathogenic Enterobacteria, which included passive surveillance in broiler and turkey farms, controls of the poultry meat destined for human consumption, and groundwater sampling. Isolates were cultured on Rambach agar and incubated overnight at 37°C. Commercial antisera were used to serotype *Salmonella* isolates (Statens Serum Institut, Copenhagen, Denmark) by slide agglutination, as described by Kauffmann–White scheme (Ewing, 1972; Guibourdenche et al., 2010). A set of 103 isolates of *S.* Infantis, which included only one sample per sampled lot, were selected for further analysis.

The antimicrobial susceptibility test was performed using a microdilution method using the Sensititre automated system with TES (Thermo Fisher Scientific Inc., Waltham, MA, United States) and the Sensititre™ EUVSEC (Thermo Fisher Scientific Inc., Waltham, MA, United States). The *Escherichia coli* strain ATCC 25922 was included as a reference and susceptibility values were interpreted using EUCAST breakpoints. A panel of 14 antimicrobials: azithromycin (2–64 μg/mL), ampicillin (1–64 μg/mL), cefotaxime (0.25–4 μg/mL), ceftazidime (0.5–8 μg/mL), chloramphenicol (8–128 μg/mL), ciprofloxacin (0.015–8 μg/mL), colistin (1–16 μg/mL), gentamicin (0.5–32 μg/mL), meropenem (0.03–16 μg/mL), nalidixic acid (4–128 μg/mL), sulfamethoxazole (8–1024 μg/mL), tetracycline (2–64 μg/mL), tigecycline (0.25–8 μg/mL), and trimethoprim (0.25–32 μg/mL) was used.

### Whole Genome Sequencing

Total genomic DNA was extracted from 103 bacterial isolates using Maxwell 16 Tissue DNA Purification Kit, according to the standard protocol supplied by the manufacturer. Total DNA was quantified with Qubit DNA HS assay (Thermo Fisher Scientific Inc., Waltham, MA, United States) and sequenced with Illumina NextSeq 500 instrument. Briefly, Nextera XT Library Preparation Kit (Illumina, St. Diego, CA, United States) was used to generate sequencing libraries, which were then sequenced in 300 cycles using NextSeq500/550 Mid Output Reagent Cartridge v2, according to manufacturer’s instructions. Paired-end 150 bp reads were generated and after demultiplexing and adapter removal the quality of reads was assessed using FastQC v0.11.5 (Andrews, 2010). The raw reads were trimmed with Trimmomatic v 0.36 (Bolger et al., 2014) using base quality parameters—Leading: 25; Trailing: 25; Slidingwindow: 20:25. Genome scaffolds were assembled using SPAdes v 3.11.1 (using parameters –k 21, 33, 55, 77; –careful) (Bankevich et al., 2012) and the scaffold quality was evaluated using QUAST v 4.3 (Gurevich et al., 2013).

The set of 103 *S.* Infantis genome paired-end sequencing reads from this study was deposited in SRA repository found under Bioproject PRJNA771355. An additional set of 160 publicly available SRA sequences of *S.* Infantis were downloaded on April 7, 2021 and processed as described below. The list of SRA sequences and associated minimal metadata set are shown in Supplementary Table 1.

### *In silico* Identification of AMR Genes, Mobile Elements, and Virulence Factors

The set of 263 genomes was characterized *in silico* using ABRicate v 1.0.1, with default settings, in conjunction with four databases, all updated on March 27, 2021 (Seemann, 2020). Specifically, PlasmidFinder (460 sequences) (Carattoli et al., 2014) was used to detect plasmid incompatibility (Inc.) groups, NCBI (5,386 sequences) (Feldgarden et al., 2020) and ResFinder (3,077 sequences) (Zankari et al., 2012) were used for identification of AMR genes and VFBD (2,597 sequences) (Chen et al., 2015) was used for detection of virulence factors. To exclude truncated gene sequences for AMR genes, a positive hit was accepted only if the% coverage of the identified gene was 100%. Mutations in *gyrase A* gene were identified using PointFinder and only known mutations were considered (Zankari et al., 2017). To predict the presence of pESI-like plasmid replicons we screened the genome assemblies for the pESI-like gene pattern proposed by McMillian et al. (2020) which included *ard*D, I1 relaxase, *sog*S, *trb*A, pESI *rep*A, pESI hypothetical backbone sequence, K88, *ybt*, *mer*A, *ipf*, and *pil*L (McMillian et al., 2020). BLAST 2.12.0 + (default parameters) was used to detect the pESI-like sequences, as described previously (McMillian et al., 2020).

### Plasmid Sequence Reconstruction

To identify the AMR genes carried on specific plasmids, we used MOB-recon tool v 3.0.0 from MOB-suite package to segregate assembly contigs into predicted plasmid sequences (Robertson and Nash, 2018). Putative plasmid sequence assemblies that contained contigs with assigned Inc. plasmid groups were selected for further analysis. An exception was made for a large putative conjugative plasmid containing between 80 and 110 kbp (Mob ID AA474) that carried MOBP relaxase and partial pESI-like pattern (see “*In silico* identification of AMR genes, mobile elements, and virulence factors”), which was considered a part of the IncFIB plasmid if, additionally, it did not contain IncI sequence. The plasmid assemblies were analyzed by ABRicate v 1.0.1 to identify AMR genes. An AMR gene present on the same contig as Inc. replicon sequence, even if not segregated into a plasmid assembly by MOB-recon, was considered a part of the plasmid.

### Multilocus Sequence Typing and Core Genome Multilocus Sequence Typing

Assembled genomes were imported into Ridom SeqSphere+ software, version 6.0.2 (Jünemann et al., 2013) and core genome multilocus sequence typing (cgMLST) profiles were assigned using the default *Salmonella enterica* task template with 3,002 core gene targets created based on EnteroBase *S. enterica* cgMLST v2 scheme,^1^ as previously described (Di Marcantonio et al., 2020). Default settings were applied for allele calling and cgMLST complex detection [complex cut-off ≤ 7 loci (Dangel et al., 2019)]. Only genomes containing ≥98% good target sequences were used in further *in silico* analyses. Minimum-spanning tree (MST) was generated by pairwise comparison of cgMLST alleles ignoring missing values. Multilocus sequence typing (MLST) analysis of the set of 103 strains sequenced in this study was performed in Ridom SeqSphere + using the Achtman *Salmonella* seven locus MLST scheme, available at http://enterobase.warwick.ac.uk/species/index/senterica. A novel MLST profile (ST-8528) was generated for strain 2020-CB-4517-1-2 by submitting the sequencing reads directly to EnteroBase.

### Phylogenetic Analysis

Core single-nucleotide polymorphisms (SNPs) of 263 *S.* Infantis strains were identified in Ridom SeqSphere + software version 6.0.2. Briefly, the assemblies were imported into the Ridom SeqSphere + and the genomes with fewer than ≥98% good target sequences were discarded. Following assignment of cgMLST profiles, the polymorphisms were identified in the target sequences of alleles from EnteroBase schema and exported as an alignment of concatenated SNPs. Indels and variants present only in the reference sequence were not included in the analysis.

Phylogenetic tree was constructed using the SNPs alignment in IQ-TREE version 1.6.9 (Nguyen et al., 2015). K3P + ASC was determined by the tool as the best suited substitution model and used for reconstruction of phylogeny. Maximum-likelihood tree was midpoint-rooted and visualized in iTOL (Letunic and Bork, 2019).

SNPs in the sequences of IncX1 and IncX4 plasmids were called with *in silico* Genotyper (ISG) version 0.16.10-3 (Sahl et al., 2015) using default parameters, with BWA-MEM version 0.712-r1039 (Li and Durbin, 2009) used as a sequence aligner and GATK version 3.9 (McKenna et al., 2010) used as an SNP caller. IQ-TREE was used to generate plasmid-based phylogenies and the trees were visualized using FigTree version 1.4.1 (Rambaut, 2010).

## Results

### Clonal Population of *Salmonella* Infantis Predominates in Local Animal Farms

**FIGURE 1:**
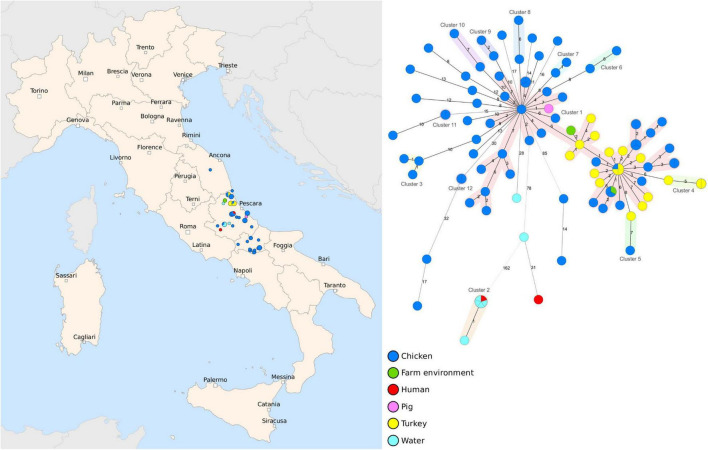
Geographic distribution and genetic diversity of *Salmonella* Infantis strains collected between 2017 and 2020. A set of 103 strains collected in Abruzzo, Molise and Marche regions were sequenced by whole-genome sequencing (WGS) and the core genome multilocus sequence typing (cgMLST) analysis was performed. The MST was generated based on 3,002 target genes, with missing values ignored, and the clusters were assigned using a cut-off of seven core genes and highlighted with different colors. The nodes are colored according to the host and the branch distances correspond to the number of discriminating loci.

In our study, we analyzed a dataset of 102 *Salmonella* Infantis isolates collected between 2017 and 2020 in the Abruzzo and Molise regions, and one strain collected in the Marche region of Italy (Figure 1 and Supplementary Table 1). MLST analysis showed that all strains, except one, belonged to ST 32 and one strain was assigned a novel ST 8528. The MST based on cgMLST showed that the majority of the strains were closely related and 54 strains isolated primarily from broiler and turkeys were assigned to the same cluster (C1) based on the single-linkage cut-off of 7 genes. The maximum distance between the cgMLST profiles in C1 was 24 loci. Other 11 clusters (C2-C12) containing an average of four genomes (min = 2, max = 6) were also identified and, on the contrary to C1, these smaller complexes often contained isolates from the same farms. Interestingly, strains isolated from groundwater and humans were more distantly related to the animal isolates, and the maximum distance of 203 core genes was observed between cluster C2 and a strain obtained from a chicken. Moreover, within C2, the human isolate was assigned the same cgMLST profile as four strains collected from groundwater.

### Genomic Characterization of *Salmonella* Infantis Population From Abruzzo and Molise

The antibiotic susceptibility test results demonstrated that over 60% of the strains were resistant to ampicillin, cefotaxime, and ceftazidime and were therefore likely ESBL producers (Supplementary Table 1 and Supplementary Figure 1). In addition, these strains (62 out of 103) often exhibited resistance to other antibiotics including quinolones, sulfamethoxazole, tetracycline, and trimethoprim.

**FIGURE 2:**
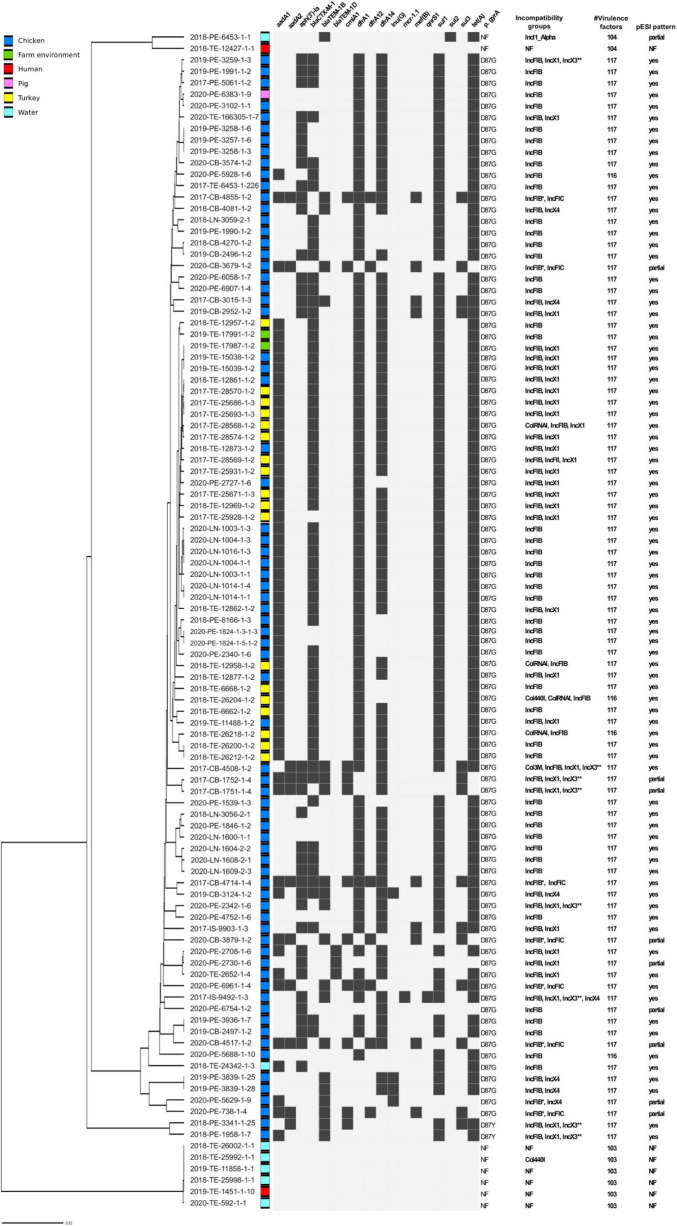
Predicted genetic traits in a set of 103 *Salmonella* Infantis strains from Italy. AMR genes detected *in silico* are shown as black boxes. NF, not found; * more than one IncFIB detected; ** located in the same contig as IncX1.

Bioinformatic analysis confirmed the presence of 62 ESBL-producing strains, all of which harbored the *bla*_CTX–M–1_ gene (Figure 2). Additionally, we detected two common broad-spectrum beta-lactamase genes *bla*_TEM–1B_ and *bla*_TEM–1D_ that in *Salmonella* spp, confer resistance to penicillins and first generation cephalosporins. The majority of isolates additionally carried multiple AMR genes, most frequently *aad*A1, *dfr*A1, *dfr*A14, *sul1* and *tet*(A), and contained a point mutation (transition A to G) in the chromosomal DNA leading to amino acid change D87G in *gyr*A associated with resistance to quinolones. MDR seen in our dataset suggested the presence of large or multiple plasmids and indeed we identified several Inc. group plasmids carried by the strains. The most frequent plasmid, found in 95 genome drafts, was IncFIB(K), which in eight isolates, was detected together with IncFIB(AP001918) and in six cases with IncFIC replicon (Supplementary Table 1). The second most common Inc. group we identified was IncX, harbored by 36 strains, that contained IncX1, IncX3, and IncX4 signatures. The sequences of IncX1 and IncX3 were always located together in the same contig, approximately 500 nt apart, but the percentage coverage and identity of the hits was higher for IncX1 (100% and 98.66% for IncX1 vs. 90.37% and 80.17% IncX3) and therefore we refer to these plasmids as IncX1.

Since previous studies associated IncFIB replicon together with *aad*A1*-dfr*A*-sul1-tet*(A) AMR pattern with the presence of pESI-like megaplasmid, we examined if the typical gene pattern proposed by McMillian et al. (2020) could be found in our set of genomes. All of the IncFIB harboring strains contained either a full or a partial set of the genes of the pESI-like pattern. In eight isolates we did not detect IncP replication origin and in six, *merA* gene coding for mercuric reductase was absent and two were missing *pil*L (pilus biogenesis) sequence. Additionally, a partial pESI pattern was detected in the isolate that harbored only IncI plasmid but the specific pESI *rep*A gene, along with seven more targets, were not identified, confirming the lack of pESI megaplasmid. The absence of the megaplasmid was associated with a lower number of virulence factors due to missing *fae*D, *fae*E, *fyu*A, *irp1, irp2* genes and yersiniabactin operon (*ybt*) (Supplementary Table 1), as shown previously (García-Soto et al., 2020).

### *In silico* AMR Analysis of Italian *Salmonella* Infantis Strains

**FIGURE 3:**
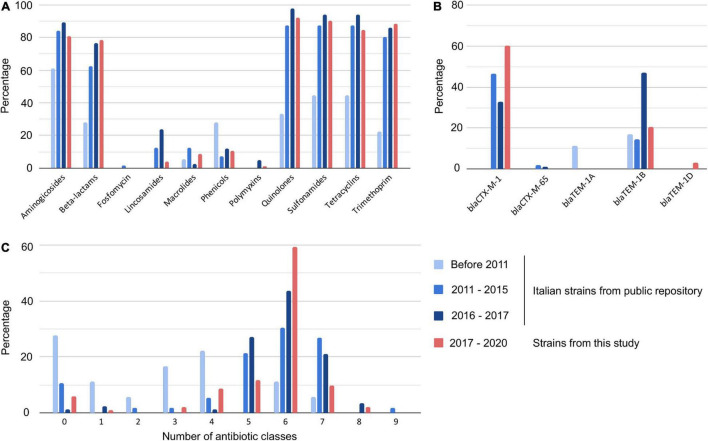
Antibiotic resistance trends of *Salmonella* Infantis collected between 2001 and 2020 in Italy. AMR genes were detected in the genomes *in silico* and assigned into antimicrobial classes. The strains were grouped into four categories and the figure panels show detected AMR traits for each of the groups. **(A)** Percentage of strains resistant to specific antibiotics, **(B)** percentage of strains carrying specific beta-lactam resistance genes, and **(C)** percentage of strains resistant to specified number of antibiotic classes.

**FIGURE 4:**
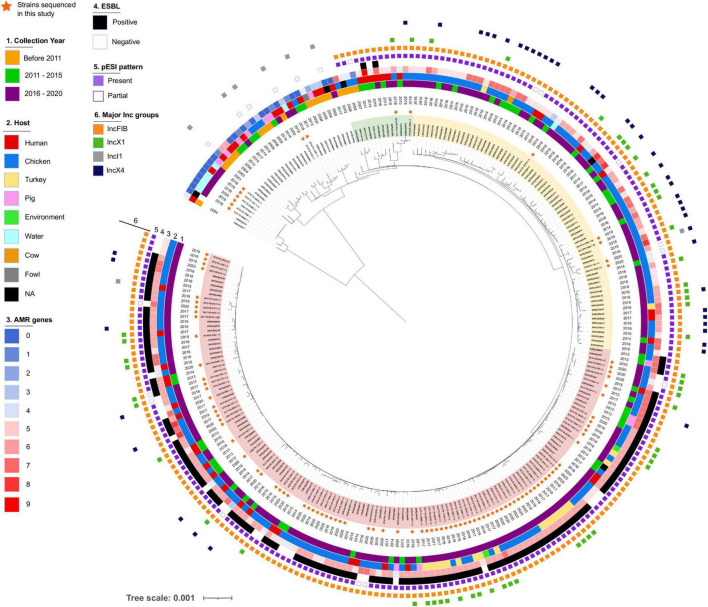
Phylogeny of *Salmonella* Infantis in Italy. *Salmonella* The maximum likelihood tree was constructed based on alignment of 1,826 concatenated core genome SNPs of 263 *S.* Infantis genomes. Strains sequenced in this study are marked with a star and three different clusters are highlighted (Cluster I in red, Cluster II in yellow, Cluster III in green).

Since our dataset contained isolates from a restricted geographical area, we analyzed additional publicly available *S.* Infantis sequences that contained strains collected in Italy between 2001 and 2017 (Supplementary Table 1). *In silico* analysis showed a lower proportion of AMR strains circulating before 2011 than those found in the following years (Figure 3 and Supplementary Table 1). In particular, the comparison of the AMR genes (or mutations) identified in the isolates collected before 2011 and between 2011–2015 demonstrated increased resistance to aminoglycosides (from 61.1 to 83.9%), beta-lactams (from 27.8 to 62.5%), quinolones (from 33.3 to 87.5%), sulfonamides and tetracyclines (from 44.4 to 87.5%), and to trimethoprim (from 22.2 to 80.4%). Based on the available WGS data, the current resistance levels of *S*. Infantis in Italy exceeded 80% of resistant isolates for aminoglycosides and trimethoprim, and 90% for quinolones, sulfonamides, and tetracycline. Moreover, a rise in the number of MDR strains was noted, with less than 20% of strains harboring five or more AMR traits before 2011 compared to more than 80% after 2011. While we detected *bla*_TEM_ genes in 27.8% of strains collected prior 2011, no ESBL-producers were identified in this group of sequences. The number of strains carrying the *bla*_CTX–M–1_ gene in the set from 2011–2015 reached 46.4% while between 2016–2017 the number was lower (32.9%). Interestingly, in the dataset from Abruzzo and Molise, the prevalence of ESBL producing strains was more than 20% higher than in the Italian strains from the public dataset from 2011–2017 and exceeded 60% of the analyzed strains. An alarmingly high proportion of human isolates carried *bla*_CTX_ genes (48%), and a similar prevalence of ESBL producers was observed in the analyzed poultry strains (47%) (Figure 4 and Supplementary Table 1).

### Phylogenetic Analysis of *Salmonella* Infantis Population in Italy

The phylogeny reconstructed using concatenated core genome SNPs of Italian *S.* Infantis strains revealed two genetic lineages, one minor, containing primarily strains isolated from groundwater in Abruzzo and one major, which was further divided into separate branches with shared characteristics (Figure 4). Isolates collected before 2011 were located in the same part of the tree and displayed a low number of AMR genes and did not express ESBL. These strains originated primarily from humans and swine and none of them harbored IncX plasmids. A partial pattern of pESI was detected in five of them and was attributed to the presence of IncI plasmids. In only two strains, isolated in 2008 and 2009, we detected a pESI-like plasmid pattern. We also observed three sparsely populated branches (Figure 4, highlighted in green) composed of strains from humans and broilers collected between 2007 and 2018 that harbored pESI like plasmid. Two of these strains expressed ESBLs encoded by *bla*_CTX–M–65_ gene along with several additional AMR genes.

Two major clusters of genomes, originating from the same branch split into two closely related populations, were widespread in Italy. Both clusters included strains carrying pESI-like IncFIB plasmids, but only one of them consisted primarily of ESBL producers (highlighted in red, Cluster I). The genetic distance between the strains contained in each cluster, based on cgMLST, were 33 (Cluster I) and 56 (cluster in yellow, Cluster II) loci. The majority of the strains sequenced in this study were placed in Cluster I, which would explain the higher genetic uniformness of this population likely caused by the restricted geographic origin of the samples. Interestingly, the distance between the most closely related genomes from these clusters was only 15 genes demonstrating close genetic relationship between the populations.

In addition to IncFIB, multiple Italian *S.* Infantis isolates harbored IncX plasmids. In particular, we noticed that IncX4 were more common within the non-ESBL producer group i.e., in the Cluster II population. Based on *in silico* data, we predicted that IncX plasmids frequently carried *bla*_TEM–1_ (Table 1 and Supplementary Figure 2). Phylogenetic trees of IncX1 and IncX4 were largely congruent with the cgMLST based phylogeny. The majority of IncX plasmids harbored by Cluster I and Cluster II isolates were grouped within their respective cluster lineages (Supplementary Figure 2). In addition to *bla* genes, IncX4 plasmids from five of the analyzed strains contained *mcr-1.1* genes responsible for resistance to colistin as previously shown by Carfora et al. (2018). Other genes identified on IncX plasmids included *aad*A1, *aad*A2, *cat*B3, *lnu*(G), and *sul3* (Supplementary Table 2).

**TABLE 1:** Predicted distribution of antimicrobial resistance genes on plasmids.

	Total	IncA/C	IncF	IncH	IncI	IncX	ND
aac(3)-IVa	2	0	2	0	0	0	0
aac(6’)-Ib-AKT	2	0	0	0	0	0	2
aadA1	131	1	105	0	5	6	14
aadA13	1	0	0	0	1	0	0
aadA2	26	0	1	0	3	2	20
aadA5	3	0	0	1	0	0	2
aph(3”)-Ib	7	0	0	2	1	0	4
aph(3’)-Ia	123	0	120	2	0	0	1
aph(4)-Ia	2	0	2	0	0	0	0
aph(6)-Id	7	0	0	2	1	0	4
blaCTX-M-1	116	0	4	0	1	0	111
blaCTX-M-65	2	0	2	0	0	0	0
blaTEM-1B	72	1	10	1	3	55	2
blaTEM-1D	3	0	0	0	0	3	0
blaTEM-1A	2	0	0	0	0	0	2
catA1	3	1	0	2	0	0	0
catB3	2	0	0	0	0	0	2
cmlA1	25	3	1	0	0	2	19
dfrA1	139	0	127	0	0	0	12
dfrA12	9	0	1	0	0	0	8
dfrA14	178	0	178	0	0	0	0
dfrA17	1	0	0	1	0	0	0
dfrA5	1	1	0	0	0	0	0
dfrA8	7	0	0	0	0	0	7
ere(A)	1	1	0	0	0	0	0
floR	2	0	2	0	0	0	0
fosA3	1	0	1	0	0	0	0
lnu(F)	1	0	0	0	0	0	1
lnu(G)	29	0	0	0	0	8	21
mcr-1.1	5	0	0	0	0	5	0
mef(B)	17	0	1	0	0	0	16
mph(A)	1	0	0	0	0	0	1
qnrB19	2	0	0	0	0	0	2
qnrS1	1	0	0	0	0	0	1
sul1	221	1	215	1	1	0	3
sul2	5	1	0	2	2	0	0
sul3	36	0	1	0	3	2	30
tet(A)	222	1	215	0	1	0	5
tet(B)	2	0	0	2	0	0	0

*ND, not determined. Increasing number of detected genes is highlighted with the red color gradient, white color marking low number of genes and dark red marking 325 large number of genes detected.*

The IncF megaplasmids contained the highest number of AMR genes including *aad*A1, *aph(3’)-*Ia, *dfr*A1, *dfr*A14, *sul1*, and *tet*(A). The ESBL gene *bla*_CTX–M–65_ was predicted to be found on the IncFIB plasmid however the location of *bla*_CTX–M–1_ gene could not be determined as it assembled as a single contig flanked by inverted repeat regions. Blastx analysis showed that this contig, which in most cases contained 4,544 nt, was additionally composed of sequences coding for MFS transporter protein and a serine resolvase. As 64 isolates that carried *bla*_CTX–M–1_ gene harbored only IncFIB replicons, it is highly probable that the gene was located on these megaplasmids, as previously shown (Alba et al., 2021).

## Discussion

In the last 10 years we have observed a sharp increase in the number of MDR strains of *Salmonella* isolated in the regions of Abruzzo and Molise. This trend has been particularly pronounced for the serovar Infantis, which has also been more frequently isolated from broilers and turkeys in the current years. In this work, we demonstrated the rise in the number of ESBL-producing *S.* Infantis collected locally, which could be attributed to an expansion of the pESI-like plasmid harboring clone which belongs to one of the two main *S.* Infantis populations circulating in Italy. These strains, in addition to the 3rd-generation cephalosporins, were frequently resistant to aminoglycosides, ciprofloxacin, tetracycline, trimethoprim, and sulfonamides, leaving limited options for the treatment of potential foodborne infections. The larger cluster of *S.* Infantis included isolates from broilers and turkeys. The presence of the same bacterial clone in the broilers from different farms could be explained by the use of the same breeding stock for re-population of the flock. Instead, the spread of *S.* Infantis between broilers and turkeys was likely a consequence of the shared farm environment.

A smaller cluster of isolates collected from groundwater and from humans was genetically diverse from the main population of *S.* Infantis found in the region. Interestingly, the isolates from groundwater were susceptible to all antimicrobials tested, unlike the strains from the major lineage. The division of *S.* Infantis population in Italy into two distinct lineages with different AMR profiles could suggest that these lineages occupy very diverse environmental niches. Since our dataset included samples collected mainly from broilers and turkeys, it is possible that the source of water contamination could be the waste from pastures and farms rearing pigs or ruminants. Indeed, while poultry is a major source of *S.* Infantis in Europe, the serovar has also been occasionally isolated from pigs and pork meat (Schroeder et al., 2015; Borowiak et al., 2017; European Food Safety Authority [EFSA] and European Centre for Disease Prevention and Control [ECDC], 2021). The national control plan for salmonellosis in Italy is focused on poultry farms, including broilers, breeding and laying hens, and fattening and breeding turkeys. Therefore, other animal species, which are not routinely screened, may be an important but overlooked source of *S.* Infantis, genetically and phenotypically diverse from the population found in poultry. Surveillance of *S.* Infantis in animals other than poultry, such as ruminants and wild birds, and characterization of their antibiotic resistome, would provide additional information about the spread of antimicrobial resistant strains in the environment and about the role of other host species in the maintenance and spread of the AMR gene pool between circulating *S.* Infantis populations.

In our study, we observed a concerningly high number of MDR strains of *S.* Infantis. *In silico* AMR analysis of strains from different years showed a sudden increase in resistance to several antibiotic classes, a change that occurred after 2011. In particular, the number of strains in which we detected genes (or mutations) conferring resistance to beta-lactams, quinolones, sulfonamides, tetracyclines, and trimethoprim doubled in the last decade. Importantly, isolates carrying genes that confer resistance to multiple classes of antimicrobials became considerably more common in the past 10 years. Comparison of our data with the national AMR levels based on the number of identified antimicrobial resistance genes showed that in Abruzzo and Molise, ESBL strains were more frequent than in the Italian public strain collection, however the percentage of MDR strains was similar. In fact, around 2011, a sharp increase in the number of strains resistant to five or more antibiotic classes was observed. It is important to stress that all *S.* Infantis isolated after 2017 analyzed in this study, except for one strain, were collected from these two regions (out of twenty regions of Italy) and therefore the increased carriage of ESBL genes may not be representative of the entire Italian *S.* Infantis population circulating in this time period. Similar trends however have been reported for *S.* Infantis in Europe, Asia, and the Americas, and have frequently been associated with the acquisition of pESI-like megaplasmid (Dionisi et al., 2011; Nógrády et al., 2012; Hindermann et al., 2017; García-Soto et al., 2020; Newton et al., 2020; Tyson et al., 2021).

The emergence of the *S.* Infantis ESBL clone in Italy was first described by Franco et al. (2016) who noted that the clone carried *bla*_CTX–M–1_ gene on the mosaic pESI-like conjugative megaplasmid that additionally rendered the bacteria resistant to multiple classes of antimicrobials, mercury, arsenic, and quaternary ammonium compounds (Franco et al., 2016). According to the authors, this plasmid, which bore a high resemblance to pESI plasmid isolated from *Salmonella* in Israel (Aviv et al., 2014), might have been present in *S.* Infantis in Italy already in 2007. Interestingly, a recent study identified isolates harboring pESI-like plasmids in strains collected in England and Wales as early as 2000 (Lee et al., 2021). Although, we did not detect pESI like signature in the strains isolated before 2011, the publicly available dataset from these years was limited to only 18 isolates and therefore was not fully representative of the strains circulating in Italy at the time. The high proportion of antimicrobial resistant strains carrying *bla*_CTX–M–1_ gene in Italian broilers after 2011 is surprising considering that the use of third generation cephalosporins is not licensed in poultry in Italy. According to a recent study, Italy currently has the second highest consumption in the EU of antimicrobials in food producing animals and the most frequently used antibiotics in broilers are penicillins and sulphonamides and in the turkeys are polymyxins, followed by penicillins and tetracyclines (Caucci et al., 2019). It is however likely that a pESI-like plasmid already carrying the *bla*_CTX–M–1_ gene was acquired and maintained in the *S.* Infantis population due to the fitness advantages conferred by the other resistance genes and virulence factors present in the plasmid while additionally rendering the strains resistant to beta-lactams (Aviv et al., 2014; Franco et al., 2016). Indeed, Alba et al. (2020) in their recent study that examined epidemiology of *S.* Infantis in Europe, suggested that acquisition of the pESI-like megaplasmid was a major factor contributing to the rapid transmission of this serovar in Europe.

We noted that only a few strains carried both *bla*_TEM–1_ and *bla*_CTX–M–1_ genes. The first were most commonly located on IncX1 and IncX4 plasmids and found mainly in the Cluster II population. The loss of *bla*_TEM–1_ genes in majority of Cluster I strains, or the loss of entire IncX plasmids, could have been triggered by acquisition of *bla*_CTX–M–1_ gene, favored due to its activity against a wider range of beta-lactam antimicrobials.

The overwhelming prevalence of *S.* Infantis resistant to multiple antibiotic classes, including ciprofloxacin and the 3rd- and 4th-generation cephalosporins in the poultry, and consequently, in poultry meat leads to an increased risk of transmission of the resistant strains to humans. The most recent report from EFSA and ECDC that included data for AMR in broilers showed that in 2018, Italy had the largest proportion in the EU of *S.* Infantis strains resistant to cefotaxime and ceftazidime (50.8%) (European Food Safety Authority [EFSA] and European Centre for Disease Prevention and Control [ECDC], 2020, 2021). Importantly, at the same time, resistance to these antibiotics in human isolates also exceeded 50%. As Italy is one of the EU members with the highest consumption of third and fourth generation cephalosporins in humans, the pressure constantly exerted on the enteric bacteria will likely lead to selection of the resistant phenotypes rendering the treatment with beta-lactam antibiotics ineffective. Moreover, the persistence of strains carrying MDR-conferring plasmids combined with the ease of horizontal transfer of mobile elements between members of Enterobacteriaceae poses a continuous threat for acquiring resistance genes by strains currently susceptible to the majority of antimicrobials. In line with the *One Health* concept, it is essential to focus on controlling of the spread of the pESI-like plasmid and the MDR strains of *S.* Infantis in poultry populations worldwide. Also, the continuous surveillance of Enterobacteriaceae and the association with specific plasmids both on national and international scales may in the future, impede the emergence and spread of particularly resistant strains. Moreover, ongoing surveillance of antimicrobial resistance within and between the ecosystems is essential to understanding the dynamics of the transmission and persistence of AMR genes at the animal-environment-human interface and to recognize the role that each of the One Health sectors play in the emergence of resistant bacterial strains. As the misuse and abuse of antimicrobials are main factors that drive the development of resistance, the monitoring of antimicrobial usage in livestock and the environment and, consequently, the adaptation of effective stewardship programs in the veterinary medicine sector, are vital in the fight against the global threat of antimicrobial resistance.

## Data Availability Statement

The datasets presented in this study can be found in online repositories. The names of the repository/repositories and accession number(s) can be found in the article/Supplementary Material.

## Author Contributions

AJ, ED, and LD conceived the study. AC, AJ, and LD designed the methodology. AA, FM, LD, KZ, and RR collected, analyzed, and/or interpreted all microbiological data. AJ performed the computational analysis. ED and GG provided funding acquisition, project administration, and resources. AJ and LD wrote the first draft. AC formally reviewed and edited the manuscript. All authors reviewed the manuscript and approved the submitted version.

## Conflict of Interest

The authors declare that the research was conducted in the absence of any commercial or financial relationships that could be construed as a potential conflict of interest.

## Publisher’s Note

All claims expressed in this article are solely those of the authors and do not necessarily represent those of their affiliated organizations, or those of the publisher, the editors and the reviewers. Any product that may be evaluated in this article, or claim that may be made by its manufacturer, is not guaranteed or endorsed by the publisher.

## Footnotes

1
http://enterobase.warwick.ac.uk
