# Supplementary figures and images for: The Current Landscape of Antibiotic Resistance of Salmonella Infantis in Italy: The Expansion of Extended-Spectrum Beta-Lactamase Producers on a Local Scale
Source: Front Microbiol. 2022 Mar 28;13:812481. doi: 10.3389/fmicb.2022.812481 (PMC8996230; doi:10.3389/fmicb.2022.812481)

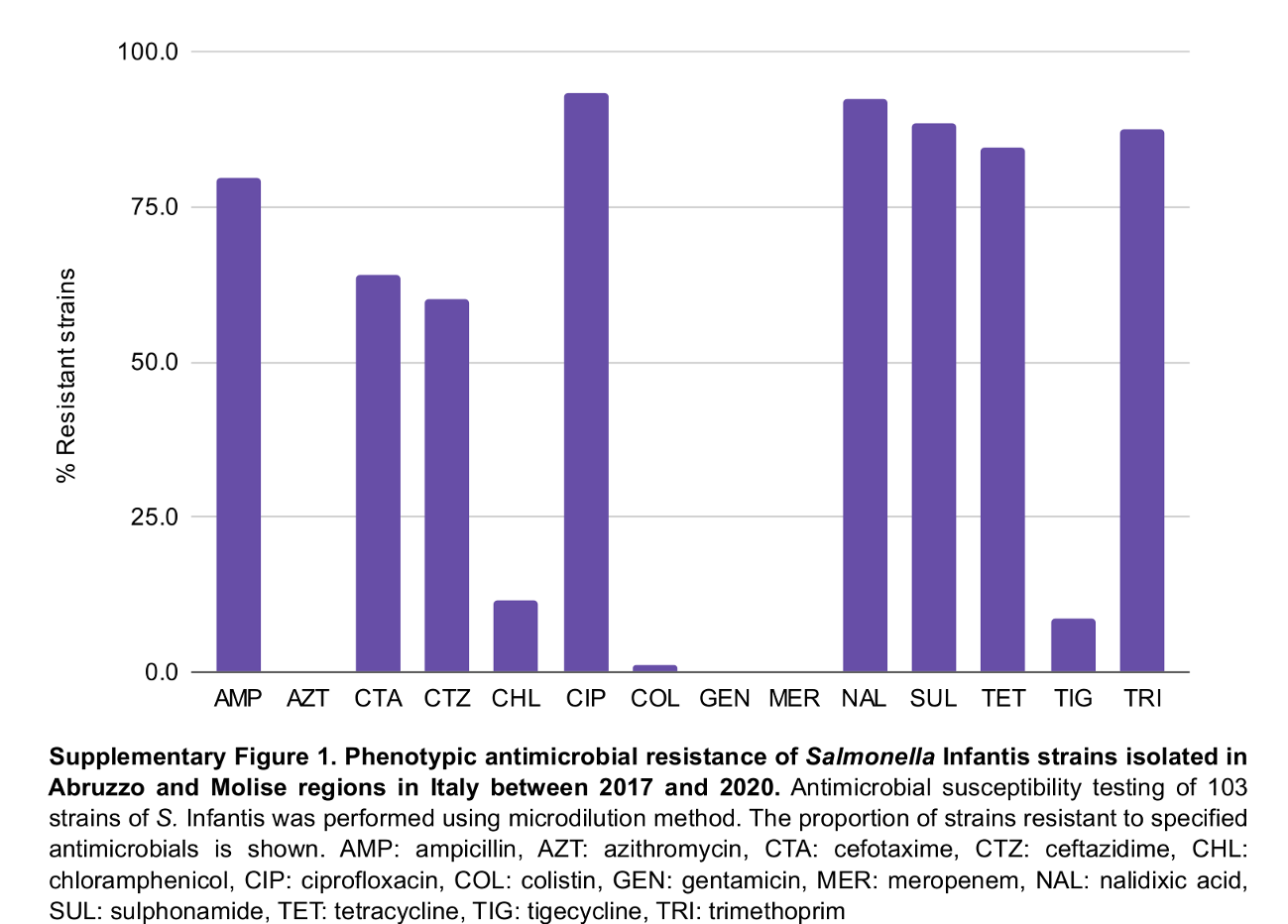

Supplement: Supplementary file 3 [file Image_1.TIF]

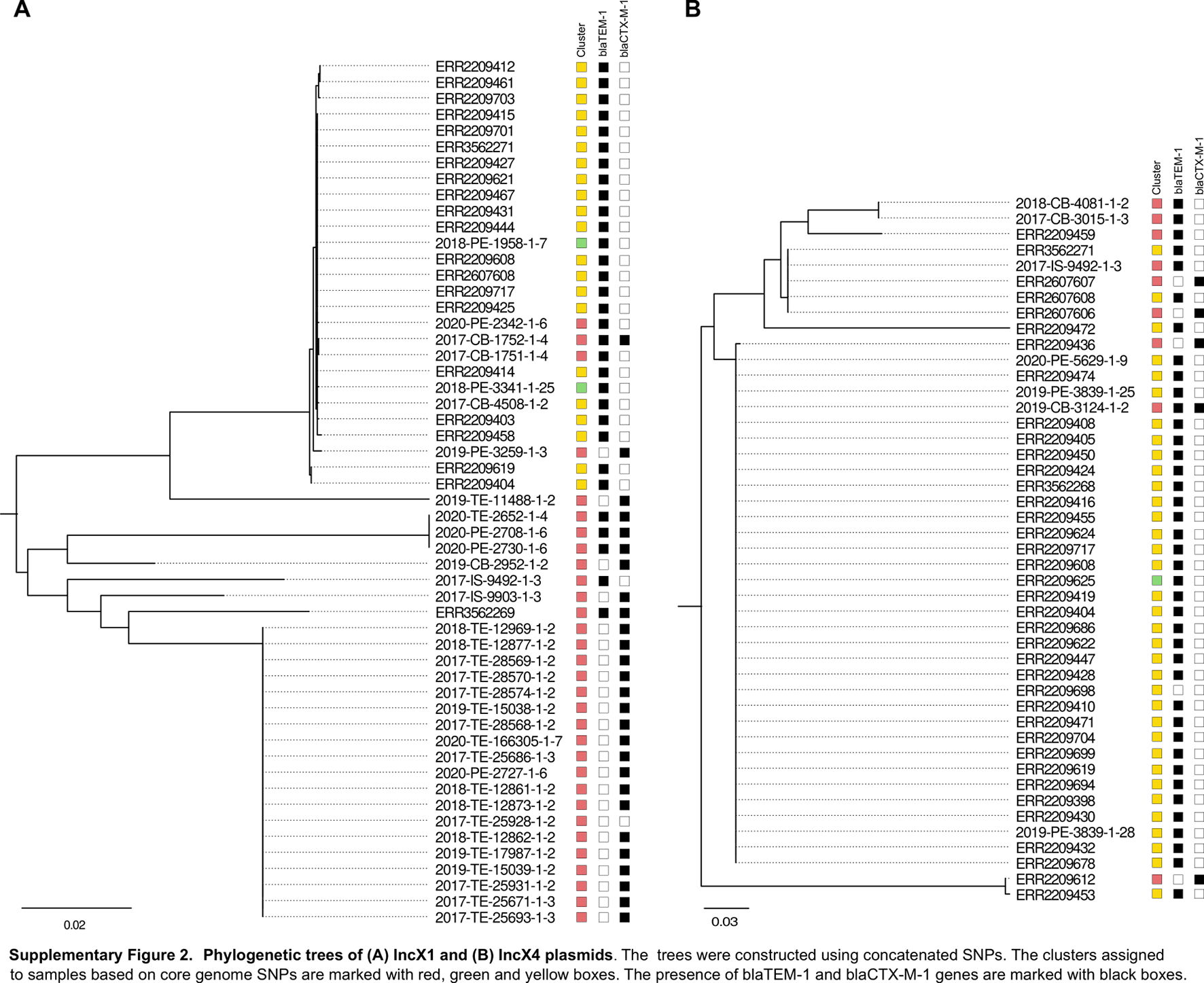

Supplement: Supplementary file 4 [file Image_2.TIF]
